# Where Does the Signal Go? Technical Challenges of CT Perfusion in Lacunar and Infratentorial Stroke

**DOI:** 10.3390/tomography12080118

**Published:** 2026-08-21

**Authors:** Nicola Morelli, Marco Spallazzi, Eugenia Rota, Marina Biondi, Davide Colombi

**Affiliations:** 1 Diagnostic Neuroradiology Unit, Guglielmo da Saliceto Hospital, Azienda USL di Piacenza, 29121 Piacenza, Italy; m.biondi@ausl.pc.it; 2 Neurology Unit, Department of Medicine and Surgery, University of Parma, 43126 Parma, Italy; mspallazzi@gmail.com; 3 Neurology Unit, San Giacomo Hospital, 15067 Novi Ligure, Italy; eugenia.rota.md@gmail.com; 4 Department of Diagnostic Imaging, Centro Diagnostico Rocca, 29121 Piacenza, Italy; colombidavide@gmail.com

**Keywords:** CT perfusion, lacunar infarction, infratentorial stroke, posterior fossa, image noise, deconvolution, down-sampling, spatial resolution

## Abstract

**Background/Objectives**: Computed tomography perfusion is widely used in acute ischemic stroke, but its performance is less reliable for lacunar and infratentorial infarcts. This technical review examines the acquisition and processing factors that influence their detectability. **Methods**: A targeted technical review of PubMed/MEDLINE, Scopus, and Web of Science was performed, focusing on acquisition, reconstruction, vascular input selection, deconvolution, filtering, spatial sampling, automated classification, posterior circulation stroke, and lacunar infarction. **Results**: Detectability depends on lesion size and contrast, posterior fossa artifacts, spatial and temporal resolution, vascular curve quality, mathematical stabilization, and automated thresholds. Larger cerebellar infarcts may remain visible, whereas brainstem, deep cerebellar, and perforator lesions are more vulnerable. Temporal maps and direct review of parametric images may reveal abnormalities absent from automated summaries. **Conclusions**: This technical review shows that computed tomography perfusion should be interpreted as a derived estimate rather than a direct representation of cerebral hemodynamics. Negative automated findings do not exclude lacunar or infratentorial infarction when clinical suspicion remains high.

## Simple Summary

Computed tomography perfusion can support acute stroke assessment, but small lacunar and posterior fossa infarcts may be missed. This technical review explains how image acquisition, reconstruction, mathematical processing, and automated thresholds can reduce the visibility of subtle lesions. Understanding these limitations may improve interpretation, prevent false reassurance from negative automated results, and support the development of more reliable imaging protocols.

## 1. Introduction

Computed tomography perfusion (CTP) has contributed to a major change in acute stroke management. Treatment selection, once governed mainly by elapsed time, can now incorporate estimates of irreversibly injured tissue and potentially salvageable parenchyma. This approach supports reperfusion therapy in selected patients presenting beyond conventional time limits and in those with wake-up stroke or an unknown time of onset [1,2,3,4,5]. Its role nevertheless depends on the clinical context. In the conventional early window, noncontrast computed tomography (NCCT) and computed tomography angiography (CTA) are generally sufficient to exclude hemorrhage, assess early ischemic change, identify an arterial occlusion, and guide reperfusion treatment. CTP is therefore not routinely required for treatment selection in this setting. Its contribution becomes more relevant when treatment depends on demonstrating a favorable tissue profile, particularly in extended windows or when onset time is unknown [1,2,3,4,5].

CTP may also assist when the presentation is atypical, clinical findings and vascular imaging are discordant, or a stroke mimic is suspected. Because seizures, migraine aura, and other nonischemic disorders may themselves produce perfusion abnormalities, the maps must be interpreted together with NCCT, CTA, vascular anatomy, and the clinical syndrome [6,7,8]. A pure motor or sensorimotor syndrome may suggest a lacunar infarct, yet the same phenotype can arise from a strategic cortical lesion, distal branch occlusion, parent artery disease, or hemodynamic impairment [9,10,11]. Clinical phenotype alone therefore provides incomplete information regarding both lesion location and the underlying vascular mechanism. Likewise, a small subcortical infarct should not automatically be equated with intrinsic small-vessel disease, as parent artery atherosclerosis and branch atheromatous disease may produce an identical imaging pattern [12].

This uncertainty is particularly relevant in lacunar and infratentorial stroke. Such lesions may produce modest attenuation changes and remain below thresholds intended to suppress noise. In the posterior fossa, beam hardening, photon starvation, partial-volume averaging, motion, and difficult vascular input selection impose additional constraints. These effects are not uniform. A large hemispheric cerebellar infarct may remain conspicuous, whereas a brainstem or deep cerebellar lesion is more vulnerable.

The final perfusion map is not a direct image of tissue hemodynamics. It is derived from a multistep processing pipeline in which subtle findings may be modified or lost. This review examines the technical basis of that process and its implications for lacunar and infratentorial stroke.

## 2. Materials and Methods

*Literature Search Strategy*. This technical narrative review was based on targeted searches of PubMed/MEDLINE, Scopus, and Web of Science covering literature published between January 2000 and May 2026. Database-specific subject headings, where available, and free-text terms were combined. Representative search combinations included (“computed tomography perfusion” OR “CT perfusion” OR CTP) AND (lacunar OR perforator OR “small subcortical infarction” OR “recent small subcortical infarct”); (“computed tomography perfusion” OR “CT perfusion” OR CTP) AND (“posterior circulation” OR infratentorial OR brainstem OR cerebellar OR “posterior fossa”); and (“computed tomography perfusion” OR “CT perfusion” OR CTP) AND (acquisition OR reconstruction OR “spatial resolution” OR “temporal resolution” OR noise OR “beam hardening” OR deconvolution OR “arterial input function” OR filtering OR down-sampling OR thresholding). Additional targeted searches addressed software performance, dual-energy CT, virtual monoenergetic imaging, arterial spin labeling, photon-counting CT, artificial intelligence, and quality control. Original studies, technical validation studies, guidelines, consensus documents, and relevant reviews were included when they directly addressed the physical principles, acquisition or processing methods, diagnostic performance, or clinical interpretation of cerebral CT perfusion, particularly in lacunar and infratentorial stroke. Publications without direct relevance to cerebral perfusion imaging or to these technical and clinical domains were excluded. Seminal publications predating 2000 were identified through the reference lists of relevant articles and included, where appropriate, to provide historical context and to describe foundational concepts underlying modern CT perfusion imaging. Study selection was qualitative and based on relevance to the technical and clinical scope of this narrative review; no quantitative evidence synthesis was undertaken.

Generative artificial intelligence was used solely for language editing and stylistic refinement. The scientific concepts, interpretations, and conclusions were developed exclusively by the authors.

## 3. From Acquisition to Perfusion Maps

**Figure 1:**
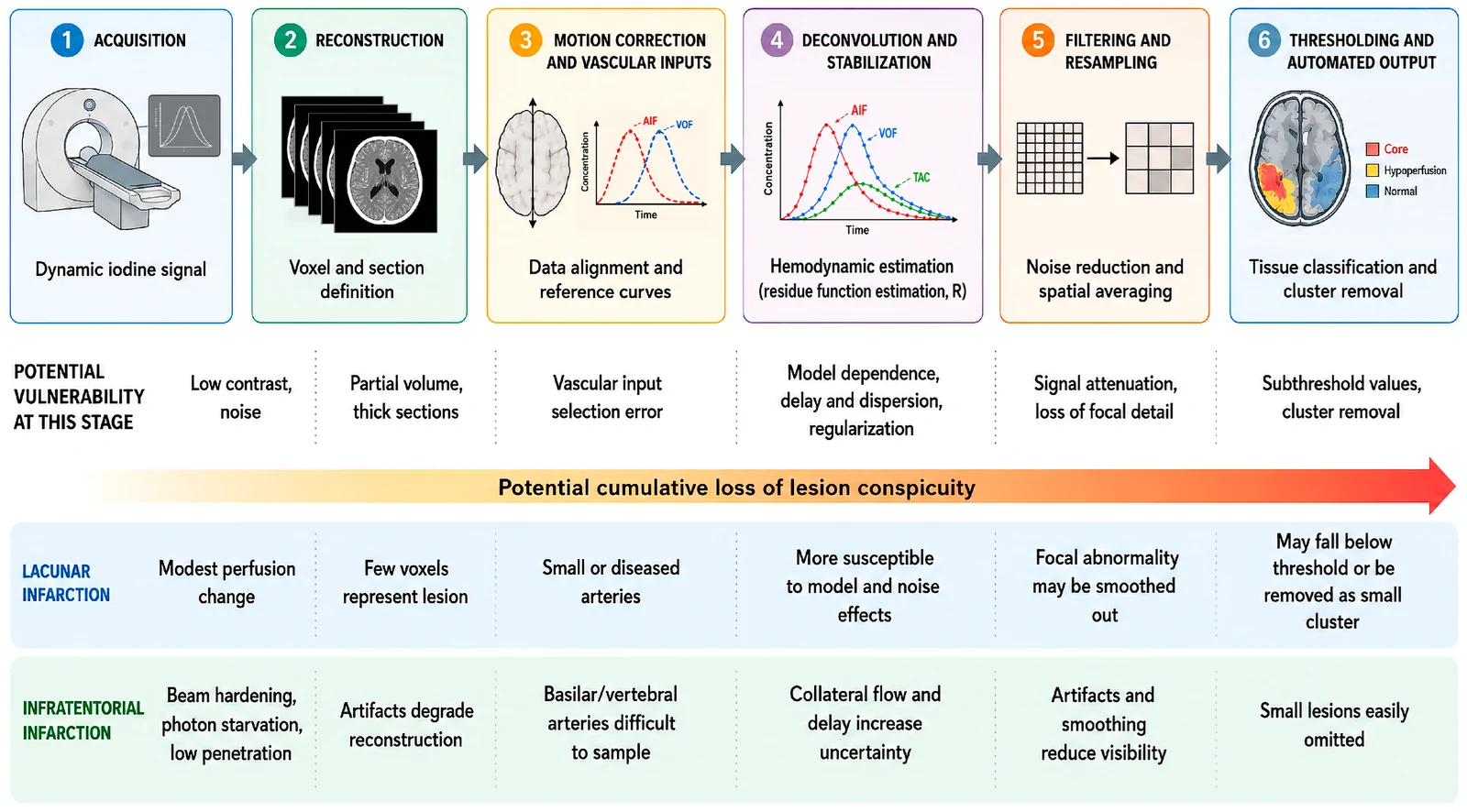
*CT perfusion processing pipeline and cumulative vulnerability of focal infarcts*. Dynamic attenuation data are transformed through acquisition, reconstruction, motion correction, vascular input selection, deconvolution, numerical stabilization, filtering, spatial resampling, thresholding, and cluster removal. Each stage improves data stability or interpretability but may also reduce lesion conspicuity. Lacunar infarcts are particularly vulnerable because of their limited volume and spatial representation, whereas infratentorial lesions are additionally affected by skull base artifacts, reduced photon penetration, and more difficult vascular input selection. The final automated output therefore reflects both tissue hemodynamics and the cumulative effects of the processing pipeline. AIF, arterial input function; VOF, venous output function; TAC, tissue attenuation curve.

CT perfusion maps are generated through a multistep process that transforms dynamic image acquisition into quantitative tissue classification. Figure 1 summarizes this sequence and highlights the stages at which limited-volume infarcts may become less detectable.

### 3.1. Signal Generation and Contrast

*Iodine attenuation and energy selection*. During CTP, repeated acquisitions measure attenuation changes produced by the passage of an iodinated bolus. After correction for baseline attenuation, these measurements generate arterial, tissue, and venous concentration curves [13,14,15]. Iodine attenuation is enhanced at lower tube voltage because a larger proportion of the X-ray spectrum lies closer to its 33.2 keV K edge. Protocols operating at 70–90 kVp can therefore improve bolus contrast, although lower-energy photons penetrate the skull base less effectively and may increase photon starvation and image noise in the posterior fossa [16,17]. Virtual monoenergetic reconstructions from dual-energy CT may partially reduce beam-hardening and streak artifacts in this region [16,18].

*Photon output and image noise*. Tube current and exposure time determine photon output. Increasing milliampere seconds raises radiation output approximately linearly, whereas quantum noise decreases according to an inverse square-root relationship. Doubling mAs therefore reduces noise by about 29%, not by one half. Because this improvement is limited, large territorial deficits may remain detectable, whereas small abnormalities with contrast close to background variation may still be obscured.

### 3.2. Perfusion Modeling and Tissue Classification

Once the dynamic signal has been acquired with sufficient contrast, it must be translated into physiological parameters.

*Tracer kinetics and the residue function*. The arterial input function (AIF) describes the contrast bolus entering the cerebral circulation, whereas the tissue concentration curve reflects how the bolus is delayed, dispersed, and retained within the local vascular bed. The *residue function*, R(t), represents the fraction of an ideal instantaneous bolus remaining within a tissue voxel at time t. It is not measured directly. According to indicator dilution theory, convolution forms the tissue concentration curve as a weighted sum of earlier arterial inputs, with R(t) providing the weights and cerebral blood flow (CBF) scaling the result. Deconvolution reverses this process and uses the measured AIF and tissue concentration curve to recover CBF × R(t), rather than R(t) alone [14,15]. Because R(0) = 1 under ideal conditions, the maximum of CBF × R(t) theoretically corresponds to CBF. In practice, temporal sampling, bolus delay and dispersion, noise, and regularization may influence the recovered function and, consequently, the derived perfusion parameters. CBF reflects the rate of blood delivery, whereas cerebral blood volume (CBV) represents the volume of blood within a given amount of tissue. Mean transit time (MTT) is the average duration of passage through the microvascular compartment and, according to the central volume principle, equals CBV/CBF. Time to maximum (Tmax) is the time at which CBF × R(t) reaches its peak and depends on both bolus delay and the deconvolution method. These effects were examined in greater detail in our previous technical review [15].

*Perfusion thresholds*. The most widely used automated paradigm identifies estimated ischemic core as tissue with relative CBF below 30% of normally perfused reference tissue. Tissue with Tmax > 6 s is commonly classified as significantly hypoperfused, and the difference between this hypoperfused volume and the estimated ischemic core is used as an operational surrogate of potentially salvageable *ischemic penumbra* [2,3,4,5,19]. These thresholds are not universal physiological boundaries. They were validated mainly in anterior circulation stroke with proximal arterial occlusion and relatively extensive perfusion abnormalities. Other platforms may produce different results because output depends on the deconvolution method, delay and dispersion correction, reference tissue selection, filtering, and software implementation [19,20,21,22,23,24]. An extended-window comparison confirmed that these processing choices can materially alter agreement with diffusion-weighted imaging (DWI) and estimated core volume [24].

*Contrast relative to background*. The signal-to-noise ratio (SNR) compares the measured signal with its variability, whereas the contrast-to-noise ratio (CNR) expresses the difference between a lesion and reference tissue relative to background variation. González reported CNR > 8 on DWI and CNR < 1 on the corresponding CT-derived CBF map, with substantially poorer delineation of lesion margins on the perfusion image [25]. These values are not universal constants, but they illustrate the limited separation between abnormal and normal tissue on derived CTP maps. The practical implication is that, when lesion-related contrast approaches background variation, subsequent processing has little margin for error.

### 3.3. Spatial Resolution, Temporal Sampling, and Dose Optimization

*Voxel size and effective resolution*. Detector collimation and primary reconstruction determine section geometry and voxel size. Thin sections reduce partial-volume averaging and preserve spatial detail, but contain fewer photons per voxel and therefore more noise. Thicker sections improve apparent uniformity at the cost of diluting lesion contrast with adjacent normal tissue. Reconstruction kernel selection also affects this balance, as sharper kernels preserve spatial detail but increase image noise, whereas smoother kernels reduce noise at the cost of potentially attenuating subtle focal abnormalities. The trade-off becomes critical when lesion diameter approaches section thickness or the finding is represented on only one or two reconstructed sections [26,27]. Primary reconstruction does not necessarily correspond to the resolution at which perfusion calculations are ultimately performed.

*Spatial down-sampling*. This processing step reduces spatial information by decreasing matrix size, combining adjacent sections, enlarging effective voxels, or calculating maps on a coarser internal grid. It reduces computational burden and voxel-level variability, but also decreases the number of samples representing a focal lesion. Data acquired with thin collimation may therefore be processed at a substantially coarser effective resolution. Down-sampling should be distinguished from interpolation and smoothing. Interpolation estimates values between sampled locations, whereas smoothing averages neighboring values to reduce local variation. Although technically different, both can alter focal detail.

*Sampling frequency and anatomical coverage*. Temporal sampling determines how frequently each anatomical level is measured. Sparse sampling may miss the true AIF peak, alter curve width, and affect derived flow and timing parameters. Acquisition must also continue long enough to include the relevant contrast passage and washout. Shuttle acquisition extends coverage along the *z*-axis by alternating table positions, but samples each anatomical level less frequently than scanners that cover the brain in a single rotation [28,29,30]. Detector row count alone does not adequately describe scanner capability, because systems with the same nominal value may differ in total detector width, rotation time, anatomical coverage, and acquisition strategy. When whole brain coverage is unavailable, slab selection or shuttle acquisition is required, whereas broader detector arrays can image the entire brain without table motion. These hardware characteristics also influence reconstruction and the behavior of proprietary software. Performance estimates and numerical thresholds should therefore be validated for the specific scanner and software used rather than transferred directly across platforms [20,21,22,23,24,28,29,30]. Spatial resolution and temporal coverage must also be balanced against photon output and radiation exposure.

*Radiation dose metrics and exposure control*. The volume CT dose index (CTDIvol) is a standardized measure of scanner output obtained in a reference phantom and does not directly represent absorbed brain, lens, or peak skin dose. The ACR–ASNR–SPR practice parameter recommends 70–90 kVp and 100–200 mAs for brain perfusion imaging [31]. Dose-check systems distinguish a *protocol-specific notification* from a *cumulative alert*. For brain perfusion imaging, the FDA proposed a cumulative CTDIvol alert value of 1 Gy, prompting formal review before proceeding [32]. This value is neither a routine target nor an absolute safety limit. Scanner-specific AAPM protocols commonly report substantially lower outputs, often in the approximate 150–300 mGy range, although dose varies with scanner architecture, acquisition mode, duration, temporal sampling, and anatomical coverage [33]. The aim is to maintain adequate photon statistics while avoiding unnecessary exposure. Automatic exposure control (AEC), including tube current modulation, may be useful in selected systems but requires scanner-specific validation.

The main stages of the processing pipeline and their practical implications are summarized in Table 1.

## 4. From Dynamic Data to Final Maps

Once the dynamic series has been acquired, the measured attenuation changes must be converted into parametric maps and automated summaries. This improves stability and interpretability, but also introduces assumptions that shape the final output.

### 4.1. Data Conditioning and Vascular Inputs

*Motion correction and anatomical masking*. Head movement causes the same voxel to contain different proportions of gray matter, white matter, vessels, cerebrospinal fluid, or bone at successive time points. Registration attempts to restore spatial correspondence, but the interpolation required for realignment may modify local curves and blur focal findings [34,35]. Brain masks exclude extracranial structures, bone, and cerebrospinal fluid, whereas vascular masks remove high-attenuation vessels that could contaminate parenchymal measurements. Excessively restrictive masking may exclude peripheral cerebellar tissue or voxels degraded by skull base artifacts. Once spatial correspondence has been restored, the software must identify the vascular curves used to model tissue perfusion.

*Arterial and venous input functions*. The AIF should show early arrival, a high peak, a relatively narrow shape, and limited partial-volume averaging. The venous output function (VOF), generally obtained from a large venous structure, is often used to scale the arterial curve and reduce amplitude error. Software may select a single voxel, a small region, several candidate arteries, or a composite curve; the method varies across platforms and versions [36,37,38]. This step can be difficult in infratentorial studies because the vertebral and basilar arteries may be small, calcified, stenotic, occluded, affected by partial-volume averaging, or degraded by artifacts from the petrous bones and clivus. An automatically selected curve may consequently have a reduced peak, excessive width, or abnormal timing. Limited *z*-axis coverage may further restrict the number of alternative vessels. Broader coverage increases the probability of including a suitable anterior circulation artery and a robust venous reference, although shuttle acquisition lowers temporal sampling at each level. Automatic curve selection should therefore be verified rather than accepted uncritically. When maps are discordant with the clinical syndrome or source images, the anatomical locations of the AIF and VOF should first be checked. Curve quality can then be assessed from baseline stability, arrival time, peak morphology, venous scaling, and completeness of bolus passage.

### 4.2. Deconvolution and Numerical Stabilization

*Deconvolution methods*. As an inverse problem, deconvolution requires the tissue response to be inferred from the measured arterial and tissue curves. Small fluctuations caused by photon noise, motion, temporal sampling, or partial-volume averaging may therefore be amplified in the estimated residue function. Deconvolution methods must balance fidelity to the measured data with numerical stability. *Singular value decomposition* (SVD) and its variants are among the most widely used deconvolution techniques in cerebral perfusion imaging [15,20,21,22]. SVD is a mathematical technique that represents the relationship between the arterial and tissue curves as a set of simpler components. Each component is associated with a *singular value* that reflects how reliably it can be recovered from the measured data. When this value is very small, the component is poorly determined and more susceptible to noise amplification during inversion. SVD estimates also remain sensitive to bolus delay, dispersion, curve truncation, and the selected regularization level. Some SVD variants, including delay-corrected and block-circulant methods, reduce sensitivity to bolus delay, whereas Bayesian approaches estimate the most probable residue function using the measured data and prior physiological assumptions. As discussed in our previous work and in independent comparative studies, Bayesian processing may improve numerical stability and agreement with DWI in selected settings, although the estimates remain dependent on the specific processing method [15,20,21,22,23,24]. All deconvolution approaches therefore require some form of numerical stabilization to prevent noise from dominating the recovered tissue response.

*Regularization, filtering, and smoothing*. Regularization uses mathematical constraints, penalties, or cutoff rules to limit noise amplification and prevent unstable or nonphysiological residue functions. In SVD-based processing, regularization may involve excluding components whose singular values fall below a selected threshold. Bayesian methods may instead impose prior assumptions that R(t) remains positive, smooth, and physiologically plausible over time. Weaker regularization preserves more low-amplitude information but allows greater noise amplification. Stronger regularization improves reproducibility but may also weaken a genuine focal change. Filtering reduces unwanted fluctuations in the data, making the underlying perfusion pattern easier to interpret. Source-image filtering modifies the dynamic data before tissue curves are generated, whereas temporal filtering reduces fluctuations along each curve and may alter peak height, width, or timing. Spatial smoothing, usually applied after map generation, averages neighboring voxel values, reducing granular noise and making broad regional patterns easier to recognize. This visual improvement should not be mistaken for greater spatial accuracy. Excessive smoothing makes lesion margins less distinct and can attenuate focal findings.

### 4.3. From Individual Parametric Maps to Automated Output

*Thresholding and cluster removal*. Perfusion values are continuous, whereas automated summaries classify tissue into operational categories such as estimated core and hypoperfusion. Following classification, connected groups of abnormal voxels below a predefined size may be removed. Cluster filtering suppresses small disconnected false-positive regions caused by noise, motion, imperfect registration, or vascular contamination, thereby improving specificity and volumetric stability. The same procedure may, however, remove a genuine lacunar or brainstem lesion when its entire representation falls below the minimum accepted cluster volume. This is not necessarily a software defect; it reflects optimization for treatment selection in large-vessel occlusion rather than for the detection of very small focal infarcts.

The main vulnerabilities specific to lacunar and infratentorial stroke are summarized in Table 2.

The technical terms most relevant to acquisition and post-processing are defined in Table 3.

## 5. Infratentorial Stroke: Why Is Detectability So Variable?

### 5.1. Anatomical and Physical Constraints

Posterior circulation stroke is anatomically heterogeneous. Large occipital or hemispheric cerebellar infarcts may produce broad, spatially coherent perfusion abnormalities, whereas pontine, medullary, and deep cerebellar lesions are generally more difficult to detect. The technical limitations discussed here therefore apply mainly to limited-volume infratentorial infarcts. Dense and irregular bone surrounding the posterior fossa preferentially absorbs lower-energy photons and alters the X-ray spectrum, producing beam hardening, photon starvation, and streak artifacts. These effects are most pronounced near the clivus and petrous bones. Because the resulting attenuation errors recur throughout the dynamic acquisition, they may distort tissue time–attenuation curves rather than merely degrade anatomical image quality. Detectability therefore depends not only on lesion size, but also on its location relative to the skull base and on the severity of local artifacts.

Dual-energy CT, through virtual monoenergetic imaging (VMI), may partially mitigate these limitations in the accompanying unenhanced CT examination. VMI uses spectral data to reconstruct images at selected monoenergetic levels, allowing a balance between gray–white matter contrast and the reduction in beam-hardening and streak artifacts. In a retrospective series of patients with suspected posterior fossa stroke, 80 keV reconstructions provided the best overall balance between artifact reduction and image quality, whereas images reconstructed at 80 and 100 keV showed potential to improve ischemia detection compared with conventional CT [16,18]. These findings relate to anatomical CT rather than dynamic CTP and should therefore not be directly extrapolated to the detection of very small infratentorial perfusion abnormalities.

### 5.2. Diagnostic Performance and Map Interpretation

These physical constraints are reflected, although not uniformly, in reported diagnostic performance. Clinical studies indicate that CTP is more sensitive than NCCT for posterior circulation ischemia but does not detect every infarct. A systematic review reported pooled sensitivity of approximately 70% and specificity close to 90%, although substantial anatomical and methodological heterogeneity limits the generalizability of these estimates [39]. Whole-brain CTP studies showed that detected infratentorial lesions were significantly larger than missed lesions [40], while more recent clinical data identified abnormalities in approximately two thirds of posterior circulation infarcts, with MTT among the most frequently positive maps [41].

**Figure 2:**
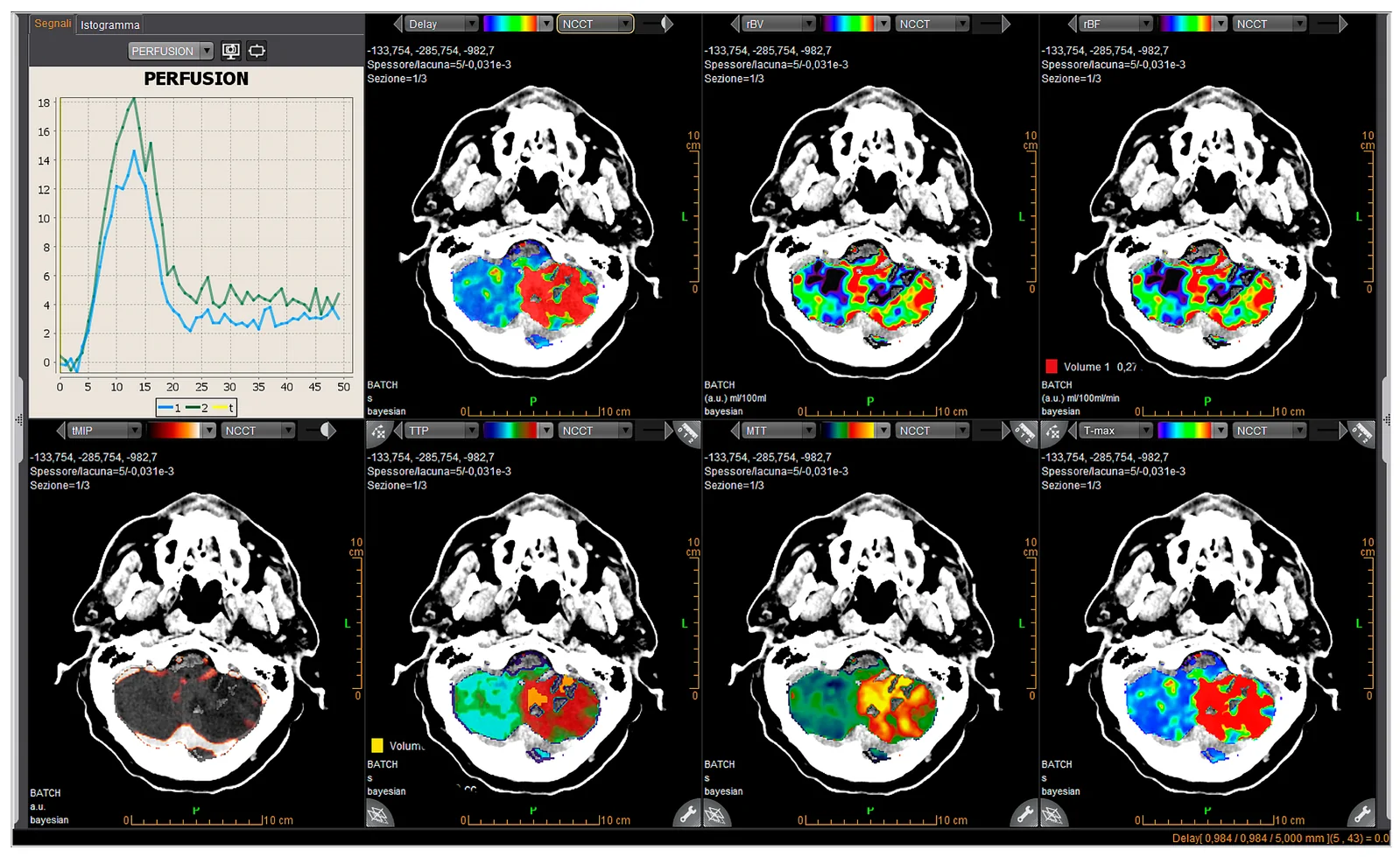
*Acute left hemispheric cerebellar infarction with a visible perfusion deficit*. The patient was examined within six hours of symptom onset for an acute vertiginous syndrome with features considered atypical for a peripheral vestibular disorder. The ***upper-left panel*** shows the time–attenuation curves obtained from the two cerebellar hemispheres. The remaining panels display color-coded perfusion maps superimposed on NCCT images. The ***upper row*** includes delay, relative cerebral blood volume (rCBV), and relative cerebral blood flow (rCBF) maps; the latter two are labeled “rBV” and “rBF,” respectively, by the software. The ***lower row*** includes temporal maximum-intensity projection (tMIP), time-to-peak (TTP), mean transit time (MTT), and time-to-maximum (Tmax; labeled “T-max” by the software) maps. CT perfusion processing was performed using a Bayesian algorithm. The examination demonstrates a perfusion deficit in the territory of the left posterior inferior cerebellar artery, more conspicuous on the temporal maps. This case illustrates that a sufficiently large and spatially coherent cerebellar perfusion abnormality may remain clearly detectable despite the technical limitations of posterior fossa imaging.

Temporal maps such as MTT, TTP, Tmax, or related delay parameters may be more sensitive than CBF and automated core maps because even a modest alteration in arrival or transit can produce a visible asymmetry. This increased sensitivity must be interpreted cautiously, as proximal stenosis, collateral pathways, cardiac output, and the deconvolution method may prolong temporal parameters without indicating irreversible injury. Figure 2 illustrates how lesion size and spatial coherence may preserve detectability despite infratentorial location.

## 6. Lacunar Stroke: How Small Is Too Small?

### 6.1. Lesion Scale and Detectability

Lacunar infarcts expose the mismatch between lesion scale and the effective resolution of the processing pipeline. Very small infarcts may be represented by only a limited number of effective voxels after reconstruction and internal resampling and may consequently fail to meet the minimum cluster size required for automated display. Clinical phenotype, imaging morphology, and vascular mechanism should nevertheless remain conceptually distinct. A pure motor syndrome may result from a small cortical embolus, whereas a recent small subcortical infarct may arise from parent artery disease or embolism. An acute lacunar infarct may also be relatively large when it involves the territory of a sizeable perforating artery. In this context, the term ‘*lacunar*’ describes the acute clinical and imaging pattern without necessarily establishing intrinsic small-vessel disease.

A systematic review of 14 studies found marked variability in CTP sensitivity for lacunar stroke, ranging from 0% to approximately 60%, with substantial heterogeneity in scanner generation, anatomical coverage, software, map selection, lesion definitions, and reference standards. Its principal conclusion was that negative CTP cannot exclude an acute lacunar infarct [42].

**Figure 3:**
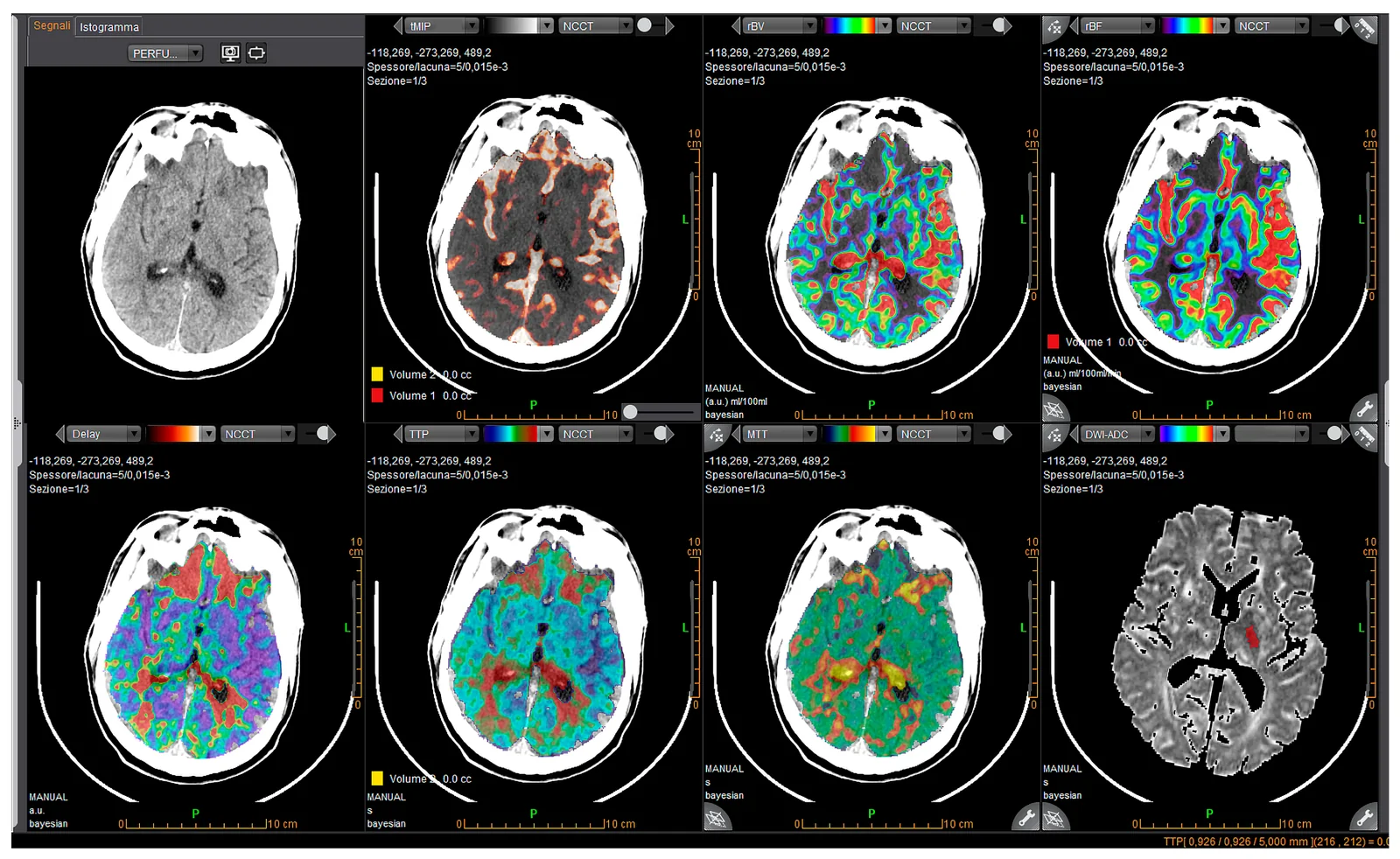
*Diffusion-positive lacunar infarct with negative CT perfusion findings*. The ***upper-left panel*** shows the automated perfusion summary superimposed on the baseline NCCT image, with no displayed ischemic core or hypoperfused tissue. The remaining panels in the ***upper row*** show the temporal maximum-intensity projection (tMIP), relative cerebral blood volume (rCBV), and relative cerebral blood flow (rCBF) maps; the latter two are labeled “rBV” and “rBF,” respectively, by the software. The ***lower row*** shows delay, time-to-peak (TTP), and mean transit time (MTT) maps, followed in the rightmost panel by an apparent diffusion coefficient map from MRI performed 20 min after CT perfusion. The ADC map demonstrates a focal acute ischemic lesion in the left internal capsule, in a perforator territory, with segmentation of cytotoxic edema using a threshold below 600 × 10^−6^ mm^2^/s. CT perfusion processing was performed using a Bayesian algorithm. No corresponding focal perfusion abnormality is identified on either the automated summary or direct review of the parametric maps, including the temporal maps. This case illustrates that a diffusion-positive lacunar infarct may remain undetected despite review of both automated output and conventional CT perfusion maps.

**Figure 4:**
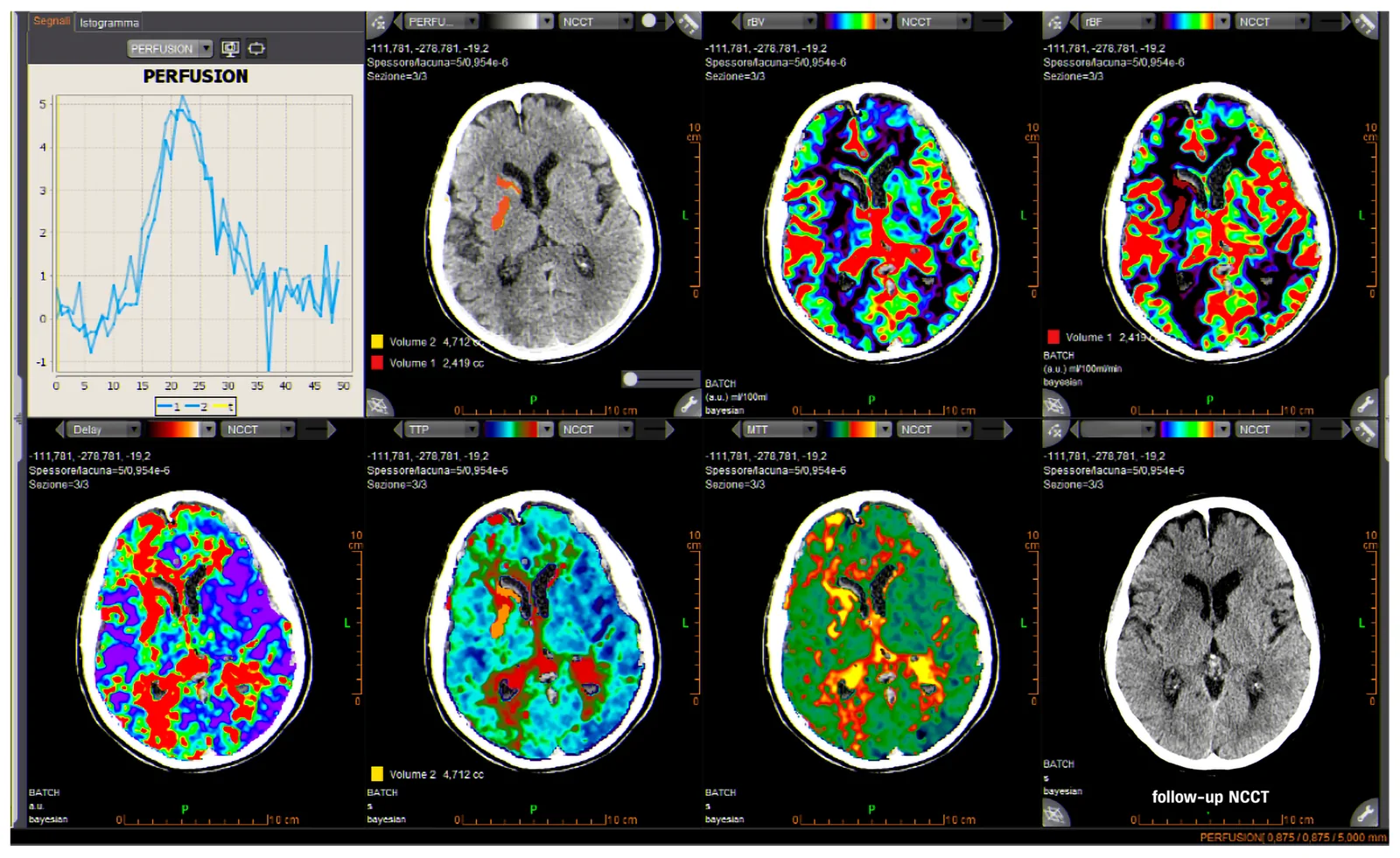
*Relatively large acute lacunar infarct with a visible CT perfusion abnormality*. This composite layout combines CT perfusion obtained at emergency department admission with follow-up NCCT performed 48 h later in the same patient. The ***upper-left panel*** shows the time–attenuation curves obtained from separate ROIs placed in the right and left cerebral hemispheres; their substantial overlap reflects a similar perfusion pattern in the two hemispheres. The adjacent panel displays the automated perfusion output superimposed on the baseline NCCT image, with the focal estimated ischemic core shown in red. The remaining panels in the ***upper row*** show relative cerebral blood volume (rCBV) and relative cerebral blood flow (rCBF) maps, labeled “rBV” and “rBF,” respectively, by the software. The ***lower row*** includes delay, time-to-peak (TTP), mean transit time (MTT), and the 48 h follow-up NCCT image in the ***rightmost panel***, which clearly demonstrates the established infarct. CT perfusion processing was performed using a Bayesian algorithm. The examination demonstrates a focal deep perfusion abnormality within a right-sided perforator territory, most conspicuous on the temporal maps. This case illustrates that a lacunar infarct may be detected when lesion size and perfusion disturbance are sufficiently pronounced, although this represents the more favorable and less common end of the lacunar spectrum.

More recent evidence suggests that perfusion information may be present even when automated core and hypoperfusion summaries are negative. In 183 DWI-confirmed lacunar infarcts with negative automated summaries, blinded visual review identified a focal abnormality in 58%. Once the DWI location was disclosed, additional subtle changes became recognizable, demonstrating the presence of subthreshold information without establishing equivalent prospective sensitivity. Perfusion-negative lesions were smaller and more frequently involved technically difficult locations [43]. Figure 3 illustrates this clinically important discordance, showing a diffusion-positive lacunar infarct without a corresponding automated perfusion abnormality. Experimental data similarly indicate that detectability increases with lesion diameter and volume, but no universal cutoff separates visible from invisible infarcts. In a model using simulated lesions and optimized thin reconstructions, detection was very poor at 5 mm and increased to more than 70% for lesions measuring 7–10 mm [44]. This finding is technically informative but does not establish 7 mm as a transferable clinical threshold, because performance also depends on lesion contrast, anatomy, acquisition, reconstruction, filtering, software, and observer. Conversely, Figure 4 illustrates a larger lacunar infarct that remains detectable on automated and parametric perfusion maps.

### 6.2. DWI and Complementary Imaging Modalities

Because CTP sensitivity varies with lesion size and processing, DWI remains the principal reference against which these findings are judged. DWI offers substantially greater lesion contrast than derived CTP maps. Reported sensitivity for acute ischemic stroke is generally above 90%, with specificity approaching 95–100%, although performance varies with imaging time, lesion size, anatomical location, and acquisition technique [45,46]. Early false-negative DWI is disproportionately associated with posterior circulation stroke, particularly with very small brainstem lesions. Oppenheim et al. reported false-negative initial DWI in 19% of posterior circulation strokes compared with 2% of anterior circulation strokes; missed lesions had a mean volume of approximately 0.19 cm^3^ and were especially frequent during the first 24 h [45]. A subsequent meta-analysis found approximately fivefold greater odds of DWI negativity in posterior circulation ischemia [46], while a later synthesis estimated DWI-negative findings in about 19% of posterior circulation events [47].

Contributing factors include limited lesion volume, relatively thick sections or interslice gaps, lower effective posterior fossa resolution, susceptibility distortion, unfavorable lesion orientation, and insufficient diffusion contrast during the earliest phase. Comparisons between CTP and DWI should therefore account for the interval between examinations and the technical quality of the MRI acquisition. A negative early DWI should not automatically classify a clinically concordant posterior fossa CTP finding as false positive.

CTA identifies arterial occlusion and characterizes collateral circulation, while CTA *source images* may also reveal parenchymal hypoattenuation. In posterior circulation stroke, however, CTP has shown greater diagnostic value than NCCT and CTA source images for detecting ischemic changes [48]. *Arterial spin labeling* provides an estimate of cerebral blood flow without contrast administration and may demonstrate hypoperfusion or delayed collateral arrival. Its lower signal-to-noise ratio and sensitivity to arterial transit delay limit direct comparability with perfusion imaging based on a contrast bolus, although acquisitions with multiple post-labeling delays can estimate transit time and reduce dependence on a single delay [49]. DWI therefore remains the principal method for lesion confirmation, CTA for vascular assessment, CTP for rapid hemodynamic evaluation within a CT protocol, and arterial spin labeling as a complementary MRI perfusion technique.

## 7. Discussion

The available evidence supports a progressive loss of lesion conspicuity rather than failure at one isolated technical stage. A focal infarct may initially produce only a modest alteration in iodine attenuation and consequently enter the processing pipeline with limited separation from normal tissue. If spatial representation or curve quality is subsequently reduced, that distinction becomes less apparent. Mathematical stabilization improves map reliability, but can also weaken low-amplitude information. By the time continuous measurements are converted into tissue categories, only a residual change may remain, and cluster rules may remove it altogether.

This sequence explains why a negative CTP examination does not have a single biological or technical interpretation. In some patients, the lesion may not produce persistent measurable hypoperfusion because of early recanalization, adequate collateral supply, or a mechanism that causes restricted diffusion without a substantial residual perfusion deficit. In others, a perfusion change may be present in the acquired data but remain too close to background variation. Motion, source-image artifacts, incomplete bolus sampling, and unsuitable vascular curves may further compromise the available information. Individual parametric maps may also retain a subtle finding that does not survive automated volumetric classification. Studies showing visually recognizable abnormalities despite negative summaries suggest that this mechanism is clinically relevant [39,40,41,42,43,44].

The distinction between source information and automated classification is therefore important. CTP software does not simply reveal a pre-existing lesion volume; it derives an estimate from a particular vascular input, mathematical model, regularization strategy, filtering approach, and set of thresholds. Identical dynamic data may consequently produce different CBF, Tmax, core, and hypoperfusion volumes when processed with different platforms or algorithms [19,20,21,22,23,24]. Terms such as “CBF core” or “Tmax lesion” remain incomplete unless the software version, deconvolution method, cutoff, and minimum displayed cluster are known.

These dependencies have practical implications for protocol design. Low kVp improves iodine contrast, but sufficient photon fluence remains necessary to control posterior fossa noise. Thin primary reconstruction preserves spatial detail, although that advantage can be lost when noise is excessive or data are later resampled onto a coarser grid. Conversely, thicker sections may produce smoother maps while diluting a focal lesion. A protocol developed to estimate a large anterior circulation core may therefore not be optimal for preserving a subcentimeter pontine or lacunar finding.

Quality assurance should extend beyond confirmation that automated processing has been completed. A practical sequence for reviewing negative or clinically discordant CTP examinations is summarized in Table 4.

Individual parametric maps remain particularly valuable when focal infarcts are suspected. Temporal parameters may reveal delayed arrival or transit even when CBF or CBV changes are modest and automated classification remains negative. Their greater apparent sensitivity should not, however, be equated with specificity. Stenosis, collateral circulation, cardiac output, and algorithmic delay sensitivity may also prolong transit measurements. The maps are therefore best interpreted together rather than as separate diagnostic tests.

This interpretative caution also applies to the reference standard. DWI remains the principal imaging reference, but an early negative result does not invariably exclude a posterior circulation infarct. A clinically concordant CTP finding should therefore be assessed in relation to symptom timing, MRI technique, and follow-up imaging rather than dismissed solely because the initial DWI is negative [45,46,47].

*Clinical interpretation of a negative CTP examination*. A negative CTP result should not override an anatomically coherent clinical syndrome. In suspected perforator-territory ischemia, recurrent stereotyped motor or sensorimotor episodes, particularly with recovery between attacks, should raise suspicion of capsular warning syndrome [50]. CTA may identify parent artery disease or an alternative large-vessel mechanism, but a negative CTA does not exclude lacunar ischemia [11,12]. In otherwise eligible patients with disabling deficits presenting within 4.5 h, a negative CTP result should not delay intravenous thrombolysis, because advanced imaging is not required for treatment selection in this time window [51]. Discordant cases should prompt reassessment according to Table 4 rather than reassurance.

*Future directions in acquisition and computational analysis*. Artificial intelligence may improve the detection of subtle perfusion abnormalities by jointly analyzing the spatial distribution and temporal evolution of the signal rather than evaluating each voxel or map independently. This approach could distinguish weak but coherent abnormalities from random noise and retain focal findings that might otherwise be attenuated by filtering or removed by cluster thresholds. It may also improve the selection and validation of vascular input functions and flag motion, incomplete bolus passage, or implausible curves before map interpretation. Models constrained by tracer kinetics may stabilize parameter estimates in noisy data, while generative methods can extract perfusion information from multiphase CTA [52,53,54,55,56]. Its principal value may therefore lie not merely in faster processing, but in more reliable recognition and preservation of subtle abnormalities. Photon-counting CT acts at an earlier stage of the imaging chain by improving the information available before perfusion analysis. Unlike conventional energy-integrating detectors, photon-counting systems register individual photons and retain information about their energy. Smaller detector elements and lower electronic noise may improve spatial resolution and gray–white matter contrast, while spectral reconstructions can provide virtual monoenergetic images and iodine maps that enhance tissue and vascular contrast and reduce artifacts near the skull base [57,58,59]. These gains could preserve subtle changes in tissue attenuation curves rather than merely sharpen anatomical images. Artificial intelligence and photon-counting CT are therefore complementary: the former may improve analysis of the acquired signal, whereas the latter may improve its quality at the source. Neither approach can fully compensate for incomplete anatomical coverage or inadequate temporal sampling, and their ability to increase dynamic CTP sensitivity for small lacunar or infratentorial infarcts remains to be demonstrated.

*Limitations of this review*. This article is a targeted technical review rather than a systematic review or meta-analysis. *First*, study selection was based on relevance to the processing pipeline and clinical question, without duplicate screening, formal risk-of-bias assessment, or quantitative evidence grading. *Second*, the available literature is heterogeneous in scanner generation, anatomical coverage, acquisition duration, radiation output, reconstruction, software, reference standard, and lesion definition. A further limitation is that important components of proprietary processing, including internal down-sampling, smoothing kernels, regularization settings, and minimum cluster volumes, are incompletely disclosed and may vary among software versions. In addition, evidence specific to infratentorial infarction remains limited, while much of the algorithmic and artificial intelligence literature derives from anterior circulation stroke. *Finally*, the clinical figures are intended to illustrate representative qualitative imaging patterns and were not used for quantitative lesion analysis or estimation of diagnostic performance. Comparisons with diffusion-weighted imaging are also influenced by the timing and technical quality of magnetic resonance imaging.

## 8. Conclusions

CTP sensitivity for lacunar and infratentorial infarcts depends on whether limited lesion-related information survives acquisition, processing, and automated classification. Large hemispheric cerebellar infarcts may remain readily detectable, whereas brainstem, deep cerebellar, and perforator lesions are more vulnerable. Temporal maps and direct review of the full set of parametric images may reveal findings absent from automated summaries. A negative automated result should therefore not exclude an acute lacunar or infratentorial infarct when clinical suspicion persists. Reliable interpretation requires awareness of the technical factors that influence lesion conspicuity throughout the CTP processing pipeline.

## Figures and Tables

**Table 1:** CTP processing pipeline and potential loss of small-lesion information.

Processing Stage	Principal Function	Main Vulnerability and Practical Implication
**Acquisition and reconstruction**	Generate dynamic attenuation data	Noise and partial-volume averaging may reduce lesion contrast; source-image quality should be checked before map interpretation.
**Motion correction and masking**	Align images and exclude unreliable voxels	Registration and restrictive masks may blur or exclude tissue; alignment and posterior fossa coverage should be verified.
**AIF/VOF selection and deconvolution**	Estimate tissue hemodynamics	Poor vascular curves and model assumptions may distort output; vessel location and curve quality should be reviewed.
**Regularization, filtering, and smoothing**	Improve numerical and visual stability	Excessive suppression may weaken focal changes; cleaner maps do not necessarily preserve more detail.
**Down-sampling and thresholding**	Reduce data complexity and classify tissue	Coarser effective resolution and fixed cutoffs may omit subtle findings; calculated-map resolution should be considered.
**Cluster removal**	Eliminate small false-positive regions	A genuine small focal lesion may be discarded; individual parametric maps should be inspected when automated summaries are negative.

**Table 2:** Factors limiting CTP detection of infratentorial and lacunar infarcts.

Technical Vulnerability	Expected Effect	Interpretative Implication	Practical Pearl
**Skull base artifacts and low CNR**	Poor lesion-to-background separation	Brainstem findings may remain inconspicuous	Interpret posterior fossa maps in clinical and vascular context
**Partial-volume averaging and down-sampling**	Dilution of focal perfusion changes	Detectability falls as effective resolution becomes coarser	Thin acquisition does not guarantee thin effective maps
**Inadequate AIF or VOF**	Distortion of perfusion estimates	Map abnormalities may reflect input-curve error	Check selected vessels before accepting volumes
**Strong regularization or smoothing**	Suppression of low-amplitude variation	Broad patterns are preserved better than focal findings	Review native or less processed maps when available
**Threshold and cluster rules**	Omission of subthreshold abnormalities	Automated output may remain negative despite a focal change	Do not use negative automated summaries to exclude focal infarction

**Table 3:** Key technical terms used in CT perfusion acquisition and processing.

Technical Parameter	Concise Definition
**Tube voltage (kVp)**	Determines maximum photon energy and influences iodine contrast and tissue penetration
**Tube current–time product (mAs)**	Reflects photon output per acquisition and affects image noise and radiation output
**Reconstruction kernel**	Mathematical filter applied during reconstruction that modifies spatial resolution and noise
**Section thickness**	Thickness of the reconstructed slice, affecting partial-volume averaging and noise
**Temporal sampling**	Frequency with which each anatomical level is measured during contrast passage
**Spatial down-sampling**	Reduction in spatial resolution through a smaller matrix, larger effective voxels, or a coarser calculation grid
**Interpolation**	Estimation of values between sampled spatial or temporal points
**Filtering**	Reduction in unwanted fluctuations in source images or time curves
**Spatial smoothing**	Averaging of neighboring map values to reduce granular noise
**Regularization**	Mathematical constraint used during deconvolution to stabilize the estimated residue function

**Table 4:** Practical review sequence for negative or clinically discordant CT perfusion examinations.

Step	Assessment	Interpretative Implication
1a	Clinical concordance: Lacunar pattern	Persistent or fluctuating motor or sensorimotor deficits without cortical signs may support ischemia in a perforator territory. Recurrent stereotyped episodes with recovery between attacks should raise suspicion of a *capsular warning syndrome*.
1b	Clinical concordance: posterior circulation pattern	Posterior circulation ischemia should be suspected when the presentation combines coherent brainstem or cerebellar findings, including ocular motor abnormalities, central nystagmus, dysarthria, dysphagia, limb or gait ataxia, crossed signs, or acute vertigo with other focal deficits. A negative automated CTP result should not override this clinical pattern.
**2**	NCCT	Review for hemorrhage, early ischemic changes, stroke mimics, and artifacts from the skull base. NCCT provides the anatomical baseline but has limited sensitivity for small posterior fossa infarcts.
**3**	CTA and source images	Assess large vessel and distal occlusions, stenoses, collateral circulation, and parenchymal asymmetry. Vascular findings should not be overridden by a negative perfusion summary.
**4**	Dynamic source data	Check anatomical coverage, motion, registration, bolus passage, curve truncation, reconstruction thickness, temporal sampling, and posterior fossa artifacts. Poor source data compromise all derived maps.
**5**	AIF and VOF	Verify vessel location, baseline stability, contrast arrival, peak morphology, curve width, and venous scaling. Implausible input curves may substantially distort perfusion estimates.
**6**	Automated output and individual maps	Compare the automated summary with CBF, CBV, delay, TTP, MTT, and Tmax maps, considering the software version, thresholds, and minimum cluster size. No single map should be interpreted in isolation.
**7**	Confirmatory imaging	Consider DWI or follow-up imaging when clinical and perfusion findings remain discordant. Further imaging should not delay time-sensitive reperfusion treatment in otherwise eligible patients.

## Data Availability

No new dataset was created or analyzed for this review. Data supporting the cited findings are available in the original publications.

## Abbreviations

The following abbreviations are used in this manuscript:

AAPM	American Association of Physicists in Medicine
ACR	American College of Radiology
ADC	Apparent diffusion coefficient
AEC	Automatic exposure control
AIF	Arterial input function
AI	Artificial intelligence
ASNR	American Society of Neuroradiology
CBF	Cerebral blood flow
CBV	Cerebral blood volume
CNR	Contrast-to-noise ratio
CT	Computed tomography
CTA	Computed tomography angiography
CTDIvol	Volume CT dose index
CTP	Computed tomography perfusion
DWI	Diffusion-weighted imaging
MRI	Magnetic resonance imaging
MTT	Mean transit time
NCCT	Noncontrast computed tomography
SNR	Signal-to-noise ratio
SPR	Society for Pediatric Radiology
SVD	Singular value decomposition
TAC	Tissue attenuation curve
Tmax	Time to maximum
tMIP	Temporal maximum-intensity projection
TTP	Time to peak
VMI	Virtual monoenergetic imaging
VOF	Venous output function
